# Supplementary material for: [68Ga]DOTATOC PET/CT Radiomics to Predict the Response in GEP-NETs Undergoing [177Lu]DOTATOC PRRT: The “Theragnomics” Concept
Source: Cancers (Basel). 2022 Feb 16;14(4):984. doi: 10.3390/cancers14040984 (PMC8870649; doi:10.3390/cancers14040984)
Supplement: Supplementary file 1 [file cancers-14-00984-s001.zip › cancers-1583293-SI.pdf]

cancers

MDPI

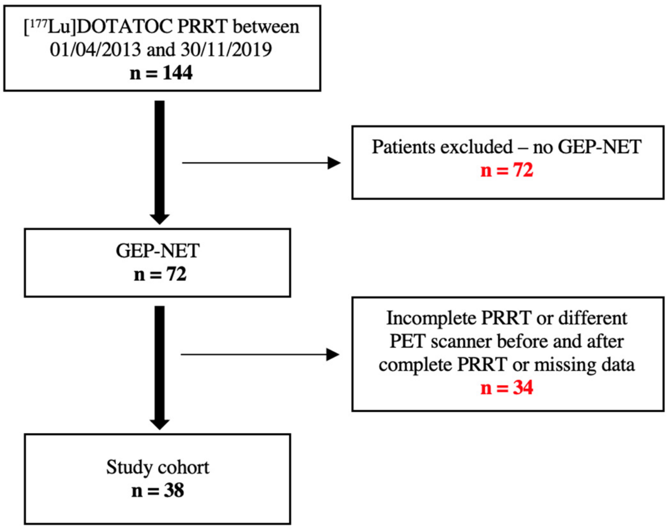

Figure S1. Patients' inclusion diagram.

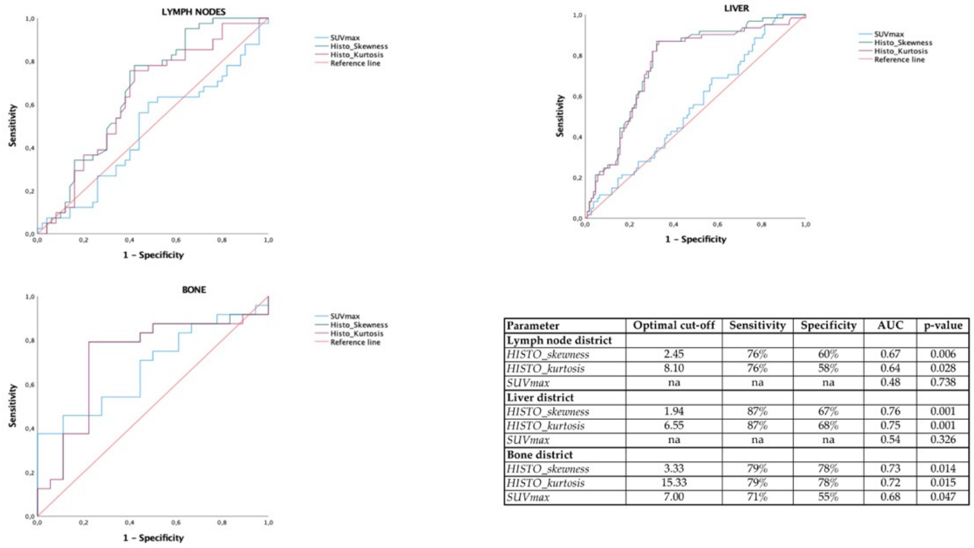

Parameter	Optimal cut-off	Sensitivity	Specificity	AUC	p-value
**Lymph node district**					
HISTO_skewness	2.45	76%	60%	0.67	0.006
HISTO_kurtosis	8.10	76%	58%	0.64	0.028
SUVmax	na	na	na	0.48	0.738
**Liver district**					
HISTO_skewness	1.94	87%	67%	0.76	0.001
HISTO_kurtosis	6.55	87%	68%	0.75	0.001
SUVmax	na	na	na	0.54	0.326
**Bone district**					
HISTO_skewness	3.33	79%	78%	0.73	0.014
HISTO_kurtosis	15.33	79%	78%	0.72	0.015
SUVmax	7.00	71%	55%	0.68	0.047

Figure S2. Receiver operating characteristics (ROC) curve analysis for HISTO_Skewness and HISTO_Kurtosis in the prediction of response to PRRT (at early PET/CT images) in terms of PD vs positive results (SD, PR, CR), for the three main districts of our cohort.

Table S1. Extracted feature full list.

**Conventional Indices**	CONVENTIONAL_Q1; CONVENTIONAL_Q2; CONVENTIONAL_Q3; CONVENTIONAL_min; CONVENTIONAL_max; CONVENTIONAL_mean CONVENTIONAL_peak; CONVENTIONAL_TLG (mL); CONVENTIONAL_RIM_min; CONVENTIONAL_RIM_stdev; CONVENTIONAL_RIM_max; CONVENTIONAL_RIM_Volume (mL); CONVENTIONAL_RIM_Volume (vx); CONVENTIONAL_RIM_sum.
**Discretized Indices**	DISCRETIZED_Q1; DISCRETIZED_Q2; DISCRETIZED_Q3;DISCRETIZED_min; DISCRETIZED_mean; DISCRETIZED_max; DISCRETIZED_peak; DISCRETIZED_TLG (mL); DISCRETIZED_RIM_min; DISCRETIZED_RIM_stdev; DISCRETIZED_RIM_max; DISCRETIZED_RIM_Volume (mL); DISCRETIZED_RIM_Volume (vx); DISCRETIZED_RIM_sum.

**First Order Features - Histogram**	Skewness ; Kurtosis; Entropy_log10; Entropy_log2; Energy.
**Shape**	Sphericity; Compacity; Volume (mL and voxels)
**Grey-Level Zone Length Matrix (GLZLM)**	Short-Zone Emphasis (SZE), Long-Zone Emphasis (LZE); Low Gray-level Zone Emphasis (LGZE); High Gray-level Zone Emphasis (HGZE); Short-Zone Low Gray-level Emphasis (SZLGE); Short-Zone High Gray-level Emphasis (SZHGE); Long-Zone Low Gray-level Emphasis (LZLGE); Long-Zone High Gray-level Emphasis (LZHGE); Gray-Level Non-Uniformity for zone (GLNUz); Zone Length Non-Uniformity (ZLNU).
**Grey-Level Run Length Matrix (GLRLM)**	Short-Run Emphasis (SRE); Long-Run Emphasis (LRE); Low Gray-level Run Emphasis (LGRE), High Gray-level Run Emphasis (HGRE); Short-Run Low Gray-level Emphasis (SRLGE), Short-Run High Gray-level Emphasis (SRHGE); Long-Run Low Gray-level Emphasis (RLGE), Long-Run High Gray-level Emphasis (LRHGE); Gray-Level Non-Uniformity for run (GLNUr); Run Length Non-Uniformity (RLNU); Run Percentage (RP).
**Neighborhood Grey-Level Difference Matrix (NGLDM)**	Coarseness; Contrast ; Busyness
**Grey Level Co-occurrence Matrix (GLCM)**	Homogeneity ; Energy; Contrast; Correlation; Dissimilarity; Entropy_log10; Entropy_log2.

Table S2. Mann-Whitney comparison of HISTO_Skewness, HISTO_Kurtosis, and SUV_{max} mean values between responders and non-responders before and after PRRT.

Features	Responders	Non-responders	p
HISTO_Skewness before PRRT	1.6 (-4.5 – 7.7)	3.5 (-0.5 – 7.7)	**< 0.001**
HISTO_Kurtosis before PRRT	9.6 (1.7 – 60.4)	18.2 (1.75 – 61.3)	**< 0.001**
SUV_{max} before PRRT	18.5 (2.1 – 91.2)	21 (1.7 – 93.5)	0.490
HISTO_Skewness after PRRT	2.5 (-1.3 – 54.1)	6 (-0.85 – 39.4)	**< 0.001**
HISTO_Kurtosis after PRRT	9.5 (1.6 – 62.1)	16.7 (0.5 – 48.9)	**< 0.001**
SUV_{max} after PRRT	13.6 (0.4 – 79.8)	15.4 (0.1 – 50.8)	**0.001**
